# Progress towards Sustainable Control of *Xylella fastidiosa* subsp. *pauca* in Olive Groves of Salento (Apulia, Italy)

**DOI:** 10.3390/pathogens10060668

**Published:** 2021-05-29

**Authors:** Marco Scortichini, Stefania Loreti, Nicoletta Pucci, Valeria Scala, Giuseppe Tatulli, Dimitri Verweire, Michael Oehl, Urs Widmer, Josep Massana Codina, Peter Hertl, Gianluigi Cesari, Monica De Caroli, Federica Angilè, Danilo Migoni, Laura Del Coco, Chiara Roberta Girelli, Giuseppe Dalessandro, Francesco Paolo Fanizzi

**Affiliations:** 1 Research Centre for Olive, Fruit Trees and Citrus Crops, Council for Agricultural Research and Economics (CREA), 00134 Roma, Italy; 2 Research Centre for Plant Protection and Certification, Council for Agricultural Research and Economics (CREA), 00156 Roma, Italy; stefania.loreti@crea.gov.it (S.L.); nicoletta.pucci@crea.gov.it (N.P.); valeria.scala@crea.gov.it (V.S.); giuseppe.tatulli@crea.gov.it (G.T.); 3 Invaio Sciences, Cambridge, MA 02138, USA; dverweire@invaio.com (D.V.); moehl@invaio.com (M.O.); uwidmer@invaio.com (U.W.); jmcodina@invaio.com (J.M.C.); phertl@invaio.com (P.H.); 4 Agrifound Italia, 50122 Firenze, Italy; glcesari@gmail.com; 5 Department of Biological and Environmental Sciences and Technologies, University of Salento, 73100 Monteroni-Lecce, Italy; monica.decaroli@unisalento.it (M.D.C.); federica.angile@unisalento.it (F.A.); danilo.migoni@unisalento.it (D.M.); laura.delcoco@unisalento.it (L.D.C.); chiara.girelli@unisalento.it (C.R.G.); giuseppe.dalessandro@unisalento.it (G.D.); fp.fanizzi@unisalento.it (F.P.F.)

**Keywords:** olive quick decline syndrome, real-time PCR, NMR metabolomic, endotherapy, sustainable development goals of the United Nations

## Abstract

*Xylella fastidiosa* subsp. *pauca* is the causal agent of “olive quick decline syndrome” in Salento (Apulia, Italy). On April 2015, we started interdisciplinary studies to provide a sustainable control strategy for this pathogen that threatens the multi-millennial olive agroecosystem of Salento. Confocal laser scanning microscopy and fluorescence quantification showed that a zinc-copper-citric acid biocomplex—Dentamet^®^—reached the olive xylem tissue either after the spraying of the canopy or injection into the trunk, demonstrating its effective systemicity. The biocomplex showed in vitro bactericidal activity towards all *X. fastidiosa* subspecies. A mid-term evaluation of the control strategy performed in some olive groves of Salento indicated that this biocomplex significantly reduced both the symptoms and *X. f.* subsp. *pauca* cell concentration within the leaves of the local cultivars Ogliarola salentina and Cellina di Nardò. The treated trees started again to yield. A ^1^H-NMR metabolomic approach revealed, upon the treatments, a consistent increase in malic acid and γ-aminobutyrate for Ogliarola salentina and Cellina di Nardò trees, respectively. A novel endotherapy technique allowed injection of Dentamet^®^ at low pressure directly into the vascular system of the tree and is currently under study for the promotion of resprouting in severely attacked trees. There are currently more than 700 ha of olive groves in Salento where this strategy is being applied to control *X. f*. subsp. *pauca*. These results collectively demonstrate an efficient, simple, low-cost, and environmentally sustainable strategy to control this pathogen in Salento.

## 1. The Phytosanitary Emergencies 

During recent years, the increase of global plant exports for agriculture and forestry has dramatically augmented the probability for phytopathogens to rapidly reach new countries and, consequently, to colonize and infect new crops and/or the same crop cultivated in another continent [1]. It should be said, however, that the introduction of pathogens and pests in a new area has also been observed in the past. For *Puccinia striiformis* f. sp. *tritici*, the causal agent of wheat yellow rust, it was established that the introduction of new populations of the pathogen into areas of wheat cultivation appear to be linked to commerce and travel over the last two centuries [2].

Pathogens can be hosted by asymptomatic potted plants, plant parts, seeds, bulbs, and timber as latent but viable cells of the pathogen that can start new infections when favorable conditions occur. The unintended introduction of new pests and pathogens in a new country poses serious risks for both cultivated crops and natural ecosystems [3]. Recent examples of newly introduced pathogens which caused severe damage to natural ecosystems include *Chryphonectria parasitica* in chestnut in southeastern Europe [4], and *Phytopthora ramorum* in oak species in the U.S.A. and in larch in the United Kingdom and Ireland [5,6]. Examples in cultivated crops include kiwifruit bacterial canker caused by *Pseudomonas syringae* pv. *actinidiae* introduced worldwide from eastern Asia in all major areas of *Actinidia chinensis* cultivation [7], and the “olive quick decline syndrome” (OQDS), caused by *Xylella fastidiosa* subsp. *pauca* introduced from Central America in Salento (Apulia, Italy) [8,9]. Dispersal of novel pathogens within an area could also be caused by many human activities not directly linked with the agricultural trade [10]. Within this context, countries with higher economic activity and population density tend to spread a greater number of biological threats [11].

Once introduced into a new area, the phytopathogens can become a permanent part of the new environment(s) depending on a series of factors such as the number of introduction events, the transmission rate of the pathogen, the density and spatial variation of susceptible host(s), the climatic conditions, and the synchronicity between host susceptibility and pathogen life cycle [12]. The climatic conditions and the complex organization of agricultural production and trade prevailing in the countries facing the Mediterranean Basin appear particularly favorable for the introduction and rapid establishment of exotic phytopathogens. This area is characterized by mild and wet winters that favor the transmission rate of pathogens and by a very rich array of cultivated and forest crops that feed the global circulation of propagative plant material [12]. The intense year-round touristic activities in this area also favor the unintended displacement and introduction of pests and pathogens from other continents [12]. Upon their introduction into a new area, the emergent pathogen should be eradicated if possible (e.g., through prompt pathogen detection, restricting the area of infection, and organized human activities) or it should be controlled according to known or new strategies to limit the disease severity and its further spread [13].

## 2. The Concept of Pathogen Control in Plant Pathology and *Xylella Fastidiosa*

The main aim in plant disease management is to reduce the economic impact of pathogens on cultivated crops [14] through control strategies that can vary according to the cropping system, the climatic features of the area, and the agronomic techniques applied to the crop [15]. Controlling a plant pathogenic bacterium does not necessarily imply its elimination from the crop but rather a significant reduction of its inoculum size that allows the crop to regularly yield [13]. In contrast, control of *X. fastidiosa* implies its elimination from the diseased plant [16], due to the quarantine status of the pathogen for Europe and the concern for its possible spread in other territories of the continent. This is a crucial and difficult task, especially when this endophytic pathogen is well-established in an area long before its first detection, as in the case of the OQDS in Salento.

Eradicating *X. fastidiosa* is made more difficult by the bacterium’s ability to colonize many wild plant species and to be effectively spread in a territory by insect vectors. Cultivars that have retained resistane to *X. f*. subsp. *pauca*, such as Leccino, have limited multiplication of the pathogen within xylem tissue but do not eliminate the bacterium from the plant [17]; consequently, growing such cultivars does not achieve the goal of eliminating *X. fastidiosa* from the area. It would therefore appear that this pathogen, when not promptly detected in a new area, has a very high probability of becoming established there and destroying the cultivated crop in few years. In contrast, satisfactory reductions of grapevine Pierce’s disease caused by *X. f.* subsp. *fastidiosa* were recently obtained with a weakly virulent strain of the pathogen [18] or with a novel bacterial biocontrol agent [19]. 

From this “control” perspective, more efforts should be made to identify strategies that significantly reduce the bacterial inoculum size within the plant across the seasons. This approach, coupled with an effective control of the insect vector(s), could allow to limit both the severity and the spread of *X. fastidiosa* in a territory, allowing crops to coexist with the pathogen similar to other bacterial diseases.

## 3. *Xylella Fastidiosa* subsp. *Pauca* in Apulia, Italy

The first record of an association of *X. fastidiosa* with olive groves showing twig and branch wilting in the Lecce province (Salento, Apulia) was reported on October 2013 [20]. The bacterium was isolated in cultures and characterized as belonging to the subspecies *pauca*, and the common name of “olive quick decline syndrome” was proposed for the disease [21]. Th main symptoms include leaf, twig and branch wilting, followed by the death of the tree. The local cultivars Ogliarola salentina and Cellina di Nardò are very sensitive to the disease. 

At the time of the first record, the disease was already spread over 8000 to 10,000 hectares of the Salento territory, corresponding to about 800,000 to 1,000,000 olive trees [22]. At that time, *X. fastidiosa* was added to the A1 list of quarantine pathogens for the European and Mediterranean Plant Protection Organization (EPPO), and, consequently, eradication measures had to be implemented, even though previous experience and the relatively widespread occurrence of the pathogen in the area made a successful outcome doubtful [23]. 

**Figure 1:**
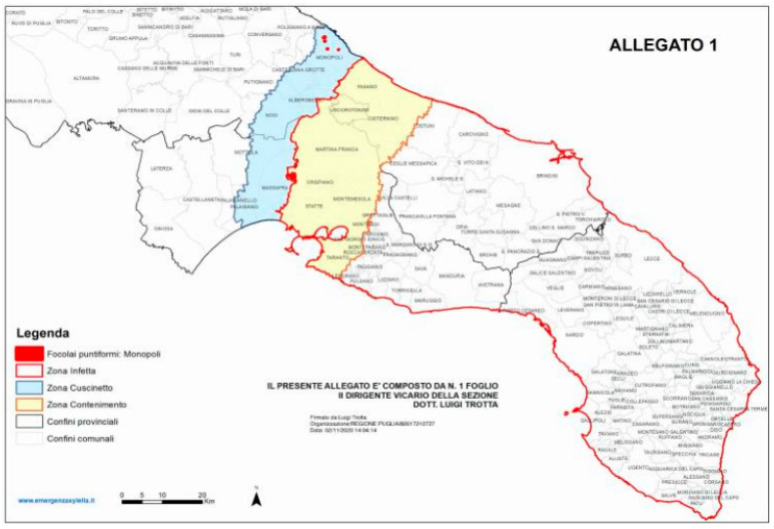
Demarcation of the “infected” (southern areas), “containment” (yellow) and “buffer” (blue) areas of Apulia according to the “Aggiornamento delle aree delimitate alla *Xylella fastidiosa sottospecie pauca ST53*”, based on the decision of the European Union 2020/1201 and on the decision of the Apulia region n° 548/2020. The “containment” and “buffer” areas are being monitored to reveal the occurrence of new olive and other plant species infected by *Xylella fastidiosa* subsp. *pauca*. Reproduced from Regione Apulia website: www.emergenzaxylella.it.

After an initial attempt to eradicate trees, an area of Salento was officially designed as “infected” and additional “containment” and “buffer” areas were established further north to monitor any occurrence of the pathogen and to prevent its spread within the region (decision of the European Union, 2016). Notwithstanding this, during the following years, *X. f.* subsp. *pauca* was reported in other olive groves of Salento in the Brindisi and Taranto provinces [24], so the extension and placement of “containment” and “buffer” areas changed during the years (decision of the European Union, 2020) (Figure 1). 

Currently, the pathogen is included into the A2 list of quarantine pathogens for EPPO, and a recent analysis has estimated that in Salento, about 6,500,000 olive trees are infected by the bacterium [25]. The monitoring plan to detect infected olive trees in the “containment” and “buffer” areas is under way, and it is carried out by the institutions of the Apulia region. 

## 4. A Control Strategy to Preserve a Multi-millennial Agroecosystem

**Figure 2:**
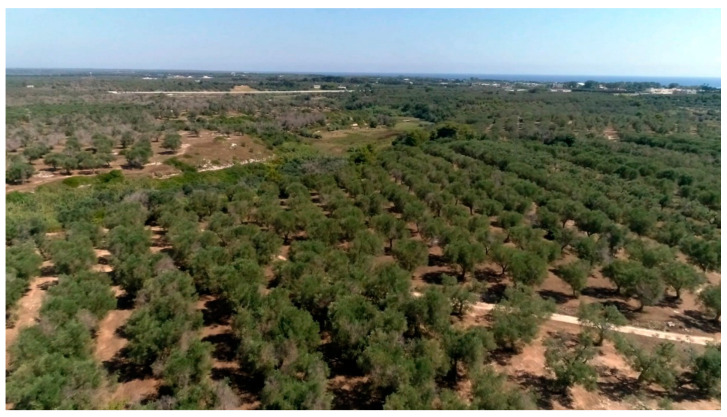
A *continuum* of olive trees that extend over kilometers characterizes the multi-millennial olive agro-ecosystem of Salento (Apulia, Italy).

The centuries-old practices employed in the olive groves of Salento, such as extensive tree management of the local cultivars and the traditional way of olive picking, characterize the whole area as a typical Mediterranean olive agroecosystem that also represents a remarkable historical, cultural, and landscaped heritage [26] (Figure 2). The local cultivars—Ogliarola salentina and Cellina di Nardò—are among the richest olive cultivars in polyphenol content and can yield an oil with a very high nutritional value [27,28]. Due to their sensitivity to the disease, the risk of losing these cultivars to disease is very high. These features of the territory prompted us to find possible field strategies that could limit both the severity and spread of the OQDS in Apulia, including identifying treatments that offer a sustainable approach to the problem and might potentially effectively limit the *X. f.* subsp. *pauca* inoculum within the plant. The preservation of such agro-ecosystem fulfills the Sustainable Development Goals (SDG) of the United Nations, namely SDG 15: “Protect, restore and promote sustainable use of terrestrial ecosystems, sustainably manage forests, combat desertification, and halt and reverse land degradation and halt biodiversity loss” (https://sdgs.un.org/goals/goal15, accessed on: 24 April 2021).

## 5. The Basic Knowledge and Criteria for Choosing an Effective Compound

In order to select a candidate formulation for establishing an effective field control strategy, an understanding of bacterial pathosystems is required for identifying potential targets in the pathogen as well as possible seasonal timings for deployment [13]. A review of the scientific literature concerning the effectiveness of substances in inhibiting the in vitro growth and biofilm formation of *X. fastidiosa* revealed that the ionomic content of the plant plays a role in the modulating the virulence of the pathogen. Zinc and copper ions, at certain concentrations, have been reported to act as effective inhibitors of bacterium’s vitality. Copper at > 0.2 mM and zinc at > 0.25 mM inhibit biofilm formation [29], whereas a sublethal concentration of zinc promotes biofilm development [30]. Zinc detoxification by the bacterium within xylematic tissue of the host plant is required for full virulence, suggesting that an additional supplement of zinc ion could inhibit xylem pathogen colonization [31]. Thus, manipulation of zinc and/or copper in the plant is a potential disease management strategy [31]. *X. fastidiosa* is characterized by an endophytic lifestyle completely within the xylem tissue. This means that the bactericidal copper compounds commonly used in topical applications to control other bacterial plant pathogens cannot reach the pathogen cells since these treatments act mainly on the plant’s surfaces and are not systemic, and therefore are not used in treating diseases caused by *X. fastidiosa*. Formulations for treating a pathogen limited to the xylem must be reliably systemic. Kirkpatrick et al. [32], referring to Pierce’s disease of grapevine, argued, “if methods could be developed to effectively deliver prophylactic or therapeutic bactericides into grapevine, this could provide a comparatively straightforward solution to a very complex disease problem”.

Thus, a suitable compound for controlling *X. fastidiosa* must be bactericidal and systemic, and ideally environmentally friendly [15] and sustainable for the olive agroecosystem. Moreover, its cost should preferably be low and its handling should be easy. Based on these criteria, we have chosen the internationally patented biofertilizer Dentamet^®^ for further evaluation. Dentamet^®^ contains a mixture of zinc (4% *w*/*w*) and copper (2% *w*/*w*) that is complexed with hydracids of citric acid through a fermentation process similar to those occurring naturally in some soil fungi. Due to its low environmental impact, this biofertilizer is also allowed for organic farming. We have (i) performed in vitro assays to test the bactericidal activity of Dentamet^®^ towards all *X. fastidiosa* subspecies, (ii) ascertained the systemicity of Dentamet^®^ within the olive xylem after foliar spraying or injection, (iii) measured the effective release of zinc and copper ions within the olive xylem, (iv) ascertained the capability of Dentamet^®^ applied by foliar spray to reduce OQDS symptoms in field trials in Salento olive orchards, (v) evaluated *X. fastidiosa* subsp. *pauca* seasonal cell concentrations in treated olive foliage by quantitative real-time PCR, (vi) ascertained the absence of copper and zinc in oil produced from treated olive trees, (vii) and considered the possibility of inciting twig resprouting in very severely damaged trees through Dentamet^®^ trunk injection.

## 6. Interdisciplinary Studies to Set Up a Sustainable Control Strategy

**Figure 3:**
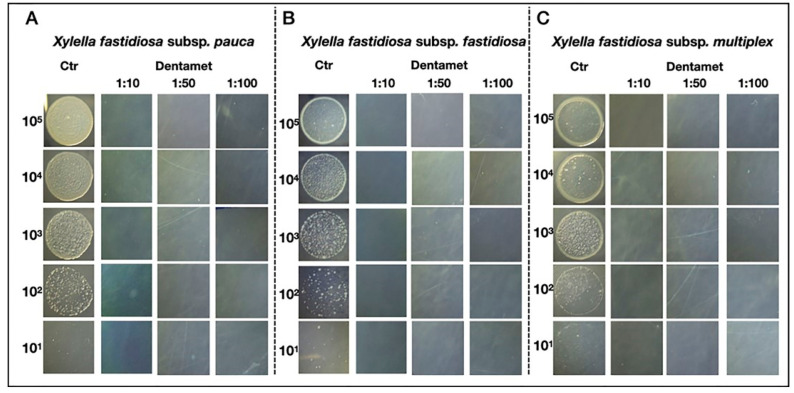
Bactericidal evaluation of *Xylella fastidiosa* subsp. *pauca* strain De Donno CFBP8402 (**A**), *X. f.* subsp. *fastidiosa* strain Temecula1 (**B**), and *X. f*. subsp. *multiplex* strain CFBP 8416 (**C**). Aliquots of 10 μL and 10-fold dilutions from 10^7^ to 10^3^ CFU mL^−1^ of each *X. fastidiosa* subspecies were spotted on PD2 medium agar plates supplemented or not with 1:10, 1:50 and 1:100 Dentamet^®^ dilutions and incubated at 28 °C. Ctr (control): *X. fastidiosa* cultures grown on PD2 medium Dentamet^®^-free. Reproduced from [34].

**Figure 4:**
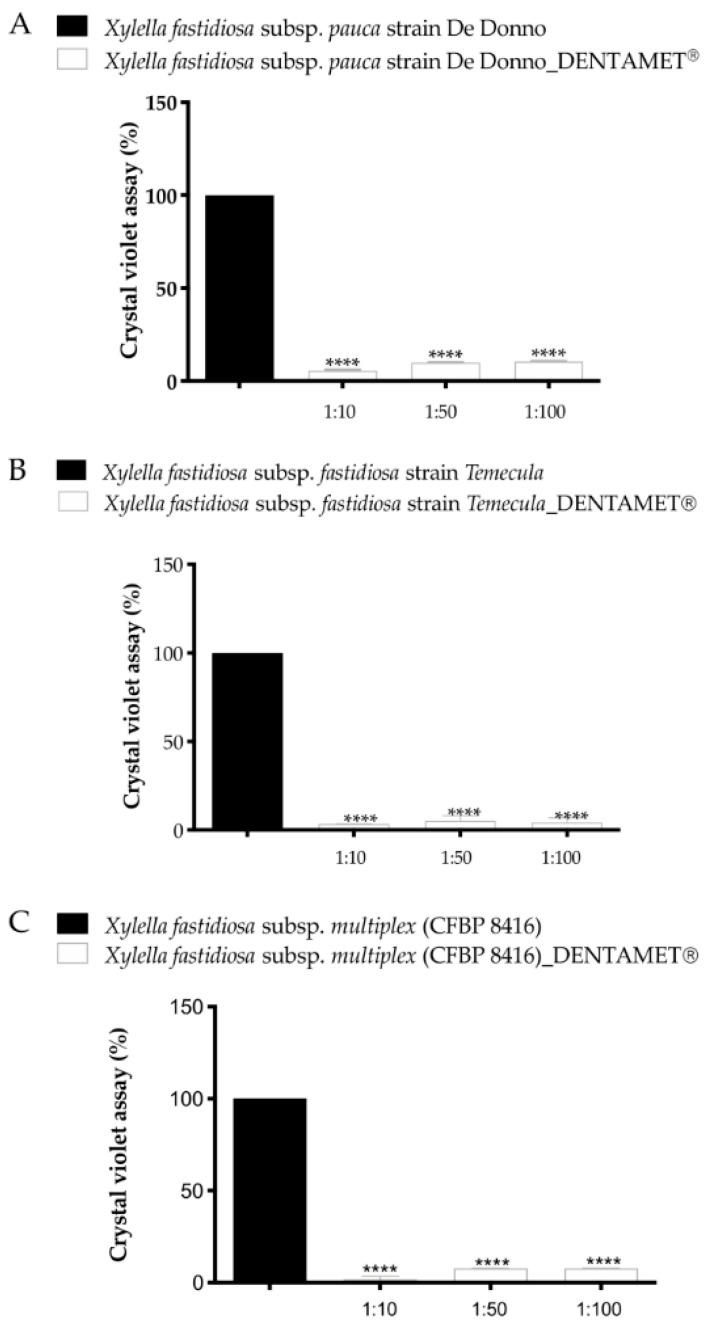
Biofilm assay 30 dpi of *Xylella fastidiosa* subsp. *pauca* (De Donno_CFBP8402) (Xfp) (**A**), and 15 dpi of *X. fastidiosa* subsp. *fastidiosa* (Temecula1) (Xff) (**B**) and *X fastidiosa* subsp. *multiplex* (CFBP 8416) (Xfm) (**C**). The ordinate axes report % of biofilm formation, the abscissa axes the Dentamet^®^ dilutions. Values are means ± SD of three independent biological replicates (*n* = 7). A statistically significant difference was obtained between Xfp, Xff and Xfm PD2 cultures (untreated control) and the same subspecies added with Dentamet^®^ dilutions (normalized with absorbance obtained by tubes without bacteria), according to one-way ANOVA, Dunnett’s test (**** *p* ≤ 0.0001 vs. Control). Reproduced from [34].

**Figure 5:**
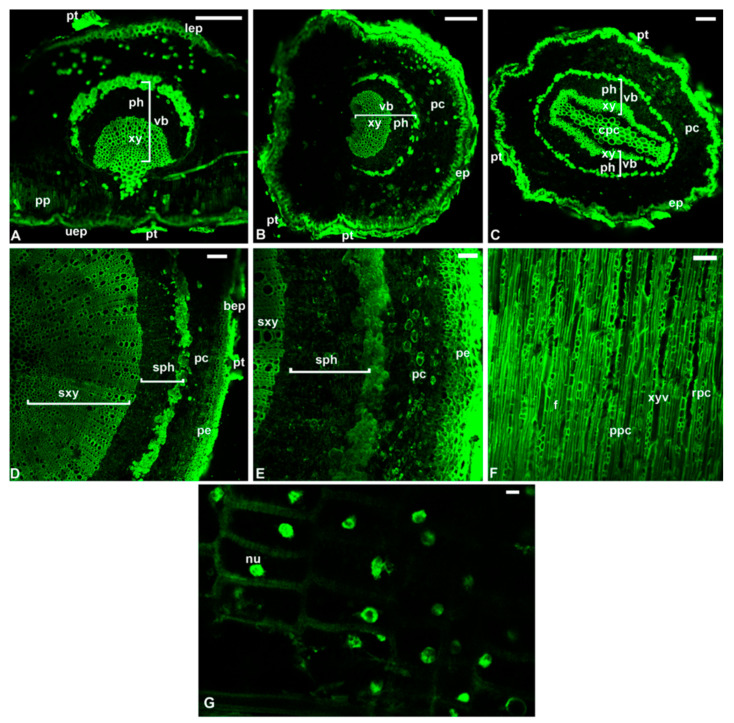
Confocal microscope images of transverse sections of leaf (**A**), petiole (**B**), peduncle of the fruit (**C**), two-year-old twig (**D**), five-year-old twig (**E**), and longitudinal tangential section of five-year-old twig (**F**) excised from a healthy olive cv. Ogliarola salentina tree sprayed with a safranin-O/Dentamet^®^ mixture. High magnification of the ray parenchyma cells (**G**). Upper epidermis (uep), peltate trichome (pt), palisade parenchyma (pp), vascular bundle (vb) with xylem (xy) and phloem (ph), lower epidermis (lep), epidermis (ep), parenchyma cells (pc), central parenchyma cells (cpc), secondary xylem (sxy), secondary phloem (sph), periderm (pe), broken epidermis (bep), xylem vessel (xyv), paratracheal parenchyma cells (ppc), fibers (f), ray parenchyma cells (rpc), nucleus (nu). Scale bars = 100 μm (**A**–**F**) and 5 μm (**G**). Reproduced from [33].

Dentamet^®^ biofertilizer showed bactericidal activity towards all the *X. f.* subsp. *fastidiosa*, *multiplex* and *pauca* strains tested, including the one isolated from olive trees showing OQDS symptoms in Apulia [33,34]. The antibacterial activity was ascertained both in broth tubes and on a bacterial culture substrate. The absence/reduction of growth was ascertained through real-time PCR. The minimum bactericidal concentration (MBC) for the biofertilizer was 400 mg/L and at 200 mg/L for zinc and copper, respectively (Figure 3). Dentamet^®^ also significantly reduced the biofilm formation in all strains tested (Figure 4). These data indicated that the biofertilizer can reduce both the pathogen vessel colonization (planktonic phase) and the biofilming phase, and justified further testing in the field. Confocal laser scanning microscopy and fluorescence quantification confirmed that Dentamet^®^ reached the olive xylem tissue either after the spraying of the canopy or injection into the trunk, demonstrating its effective systemicity. Fluorescent staining of the xylem tissue of the leaf, leaf petiole and of two- and five-year-old twigs clearly showed the migration of the biofertilizer from the entry point (Figure 5). The same tissue samples were also assessed for zinc and copper release through coupled plasma atomic emission spectroscopy, and the ions were effectively detected and quantified in the olive tissue [33]. These data indicated that the biofertilizer reaches the xylem tissue where *X. f.* subsp. *pauca* lives and multiplies.

For the field trials, three representative olive farms all located in Salento (Lecce province) in municipalities characterized by a severe occurrence of OQDS were selected, namely Veglie, Cannole and Galatone. The occurrence of *X. f.* subsp. *pauca* was ascertained on all farms before starting the trial. All olive groves were managed according to the traditional cultivation technique usually carried out in Salento: no irrigation, no regular pruning or soil fertilization, control of the main pests, free vase training system, and ample spacing between the trees. Dentamet^®^ was applied by foliar spray, i.e., by nebulization on the olive crown using an atomizer, at 3.9 kg/ha, once per month from April to September. 

An initial three-year trial was established beginning April 2015 at Veglie to assess the potential of Dentamet^®^ in reducing the impact of *X. f*. subsp. *pauca* to the local olive cultivars Ogliarola salentina and Cellina di Nardò. Subsequent trials performed at Cannole and Galatone during 2019 represented a mid-term evaluation of the pathogen control effectiveness, having started the regular application of Dentamet^®^ in those olive groves during the previous four and three years, respectively. 

A significant reduction of both field symptoms (i.e., twig wilting) and pathogen concentration within the leaves were observed at Veglie. It worth noting that during the study, apart from *X. f*. subsp. *pauca* infection, the trees faced some adverse climatic events such as frost in January 2017 and a severe heat wave during summer 2017 [33,35]. It was observed that at least 50 to 60% of the canopy should be present in the tree for satisfactory entry of Dentamet^®^ into the xylem tissue. No zinc and copper residues were found within the oil obtained from trees that received the biofertilizer over three years. 

**Figure 6:**
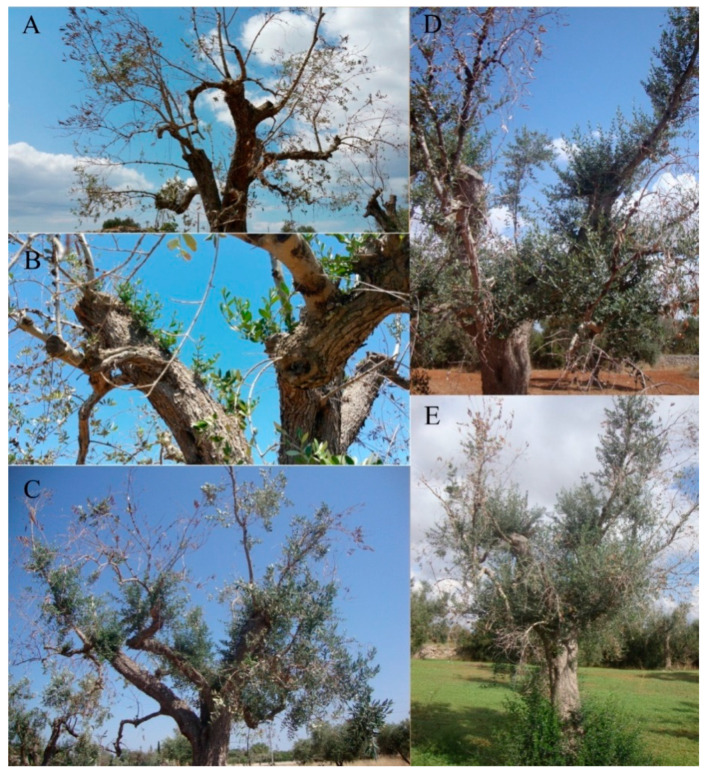
Recovery of a single Ogliarola salentina olive tree severely damaged (more than 80% of the canopy dead) by *Xylella fastidiosa* subsp. *pauca* grown in Galatone (Lecce province) after trunk injection with Dentamet^®^ from April 2017 to July 2017. (**A**) The tree before treatment. (**B**) Initial resprouting in May of shoots from the main tree branches as observed 20 d after treatment. (**C**) New shoots continued to grow during June. (**D**) At the end of July, part of the canopy was developed. (**E**) Despite the drought occurring during summer 2017, in September, the tree showed a number of suckers as well as part of the canopy. Reproduced from [33].

Finally, extensive twig resprouting in severely attacked trees was observed upon endotherapy performed at the base of the tree using disposable syringes and different doses of the biocomplex (Figure 6) [33].

In the mid-term evaluation, the tree ages ranged from 22 to more than 70 years. The traditional cultivars Ogliarola salentina and Cellina di Nardò were cultivated in all farms. Leccino trees were also present in the Galatone farm. Dentamet^®^ efficacy was evaluated either through the recording of new wilted twigs in the spring (March), summer (July), and autumn (October), or through quantitative real-time PCR performed in the same seasons on 41 trees by following the procedures established by EPPO. 

A trend that indicates a reduction of the field symptoms during the year in both farms and for all cultivars was observed. The number of wilted twigs were higher in March and decreased in July and October. Ogliarola salentina and Cellina di Nardò cultivars were confirmed to be more sensitive to *X. f*. subsp. *pauca* than Leccino. At the end of the season, just before the harvest time, only a few new wilted twigs per tree were recorded for all cultivars in both farms (i.e., a maximum of one to two twigs for the Ogliarola salentina trees). 

**Figure 7:**
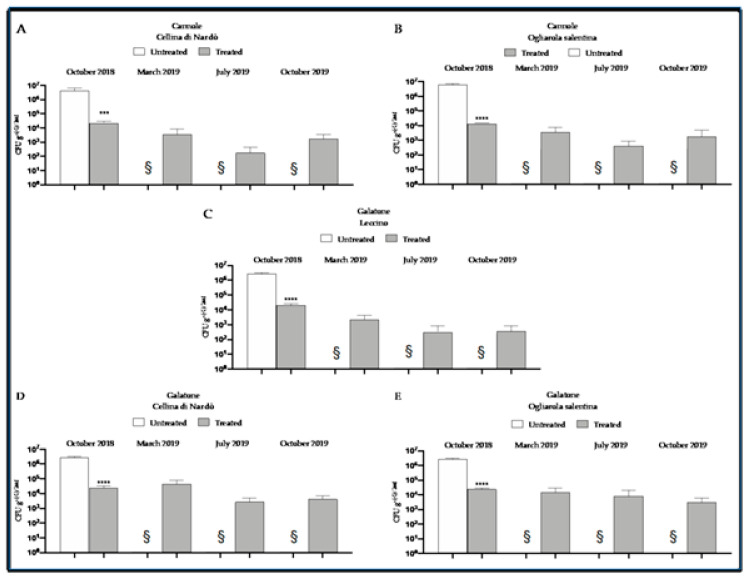
*Xylella fastidiosa* subsp. *pauca* DNA concentration, expressed in CFU equivalents g−1 of leaf, determined for untreated plant (assessed as time 0 control) and Dentamet^®^-treated cultivars Cellina di Nardò and Ogliarola salentina, at Cannole and Galatone (assessed in 2019). § Untreated control plants dead in 2019, so the absence of concentration data is indicative of plant death. (**A**,**B**) Graphical representation of the bacterial concentration over time of Cellina di Nardò and Ogliarola salentina at Cannole. (**C**–**E**) Graphical representation of the bacterial concentration over time of Leccino, Cellina di Nardò and Ogliarola salentina at Galatone. Bacterial concentration is expressed in CFU equivalents g−1 of leaf (**** *p* ≤ 0.0001 vs. controls). Reproduced from [34].

Quantitative real-time PCR analyses demonstrated a decrease in the bacterial concentration for all cultivars and both farms in July and in October following Dentamet^®^ treatments that started at the beginning of April. The mean bacterial concentration in March was 1.2 × 10^4^ CFU g^−1^, which decreased to 2.1/2.2 × 10^3^ CFU g^-1^ in July and October, respectively). Comparison of the three cultivars located at Galatone indicated that Leccino trees had a lower bacterial concentration (mean of 9.0 × 10^2^ CFU g^−1^), in comparison to Cellina di Nardò (1.7 × 10^4^ CFU g^−1^) and Ogliarola salentina (8.7 × 10^3^ CFU g^−1^) (Figure 7). The comparatively healthy status of the Dentamet^®^-treated trees was confirmed by their yield, with a production estimated at about 18 to 23 Kg of olives per tree recorded in autumn 2019 in the farms of Galatone and Cannole, whereas the untreated trees were completely wilted [34]. These results further supported the conclusions from the first study and clearly showed that *X. f.* subsp. *pauca* can be controlled in the olive groves not completely damaged by the bacterium and that a coexistence with the pathogen is possible.

It is worth noting that the farms where the mid-term evaluation was carried out are located in areas severely attacked by the bacterium and are surrounded by completely withered olive groves.

## 7. The Reprogramming of Metabolic Pathways in the Treated Trees

**Figure 8:**
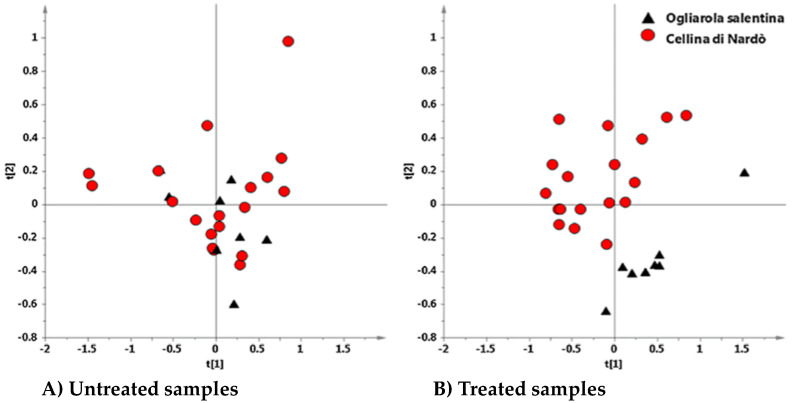
^1^H-NMR fingerprinting in combination with unsupervised principal component analysis score plots for Ogliarola salentina and Cellina di Nardò olive cultivars. (**A**) Dentamet^®^-untreated samples; (**B**) Dentamet^®^-treated samples. Reproduced from [39].

Among the analytical techniques available for the detection of molecules involved in the plant-microbe interaction, metabolomics coupled with mass spectrometry (MS) and nuclear magnetic resonance (NMR) analyses could offer several advantages for obtaining an overview of all small metabolites, and for obtaining robust, high-reproducible, spectral data [36]. The combination of NMR and chemometrics can be useful to characterize metabolic patterns in plants under stress conditions, such as pathogen infections, and to understand plant physiology, which is pivotal for future applications in disease control [37,38]. Non-targeted ^1^H-NMR fingerprinting, in combination with unsupervised (PCA) and supervised pattern recognition techniques (in particular, orthogonal partial least squares-discriminant analysis), was used for the first time by Girelli et al. [39] in order to analyze the response of naturally infected olive trees cultivars Ogliarola salentina and Cellina di Nardò, to the Dentamet^®^ treatments. In particular, the work focused on the preliminary short-time effect on the metabolic profiles of the susceptible cultivars, which showed a different incidence and severity of disease before the treatments. Interestingly, unsupervised principal component analysis (PCA) showed, only in the case of Dentamet^®^-treated samples, a clear partition of data, with a good grouping according to the original cultivar (Ogliarola salentina and Cellina di Nardò) observed (Figure 8a). On the other hand, the untreated samples submitted to PCA analysis showed no differences in the metabolomic profiles of the two cultivars (Figure 8b). These results seemed to suggest that the effect of the presence of a pathogen, such as *X. f.* subsp. *pauca* on the metabolic profile of naturally infected plants samples, could be predominant with respect to the differences normally observed among the olive cultivars. The effect of the biocomplex treatment resulted into specific involvement of xylematic polyphenols and carbohydrates for each of the two studied cultivars. In particular, treated Cellina di Nardò trees showed a higher polyphenols content, whereas treated Ogliarola salentina trees showed a higher sugar content.

**Figure 9:**
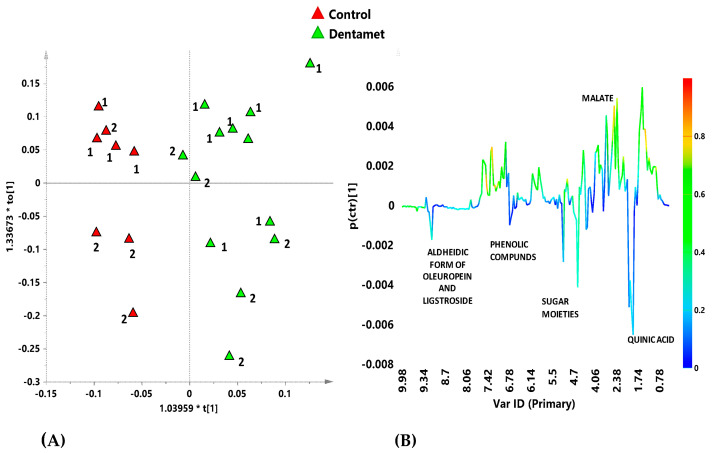
(**A**) Orthogonal partial least squares-discriminant analysis (OPLS-DA) score plot for Ogliarola salentina treated with Dentamet^®^ (green triangles) and untreated (control) (red triangles) leaf extracts samples (1 + 3 + 0; R2X = 0.613; R2Y = 0.815; Q2 = 0.418); (**B**) S-line plot for the model, indicating molecular components responsible for the class separation. The corresponding predictive loadings are colored according to the correlation scaled loading [p(corr)]. Reproduced from [40].

**Figure 10:**
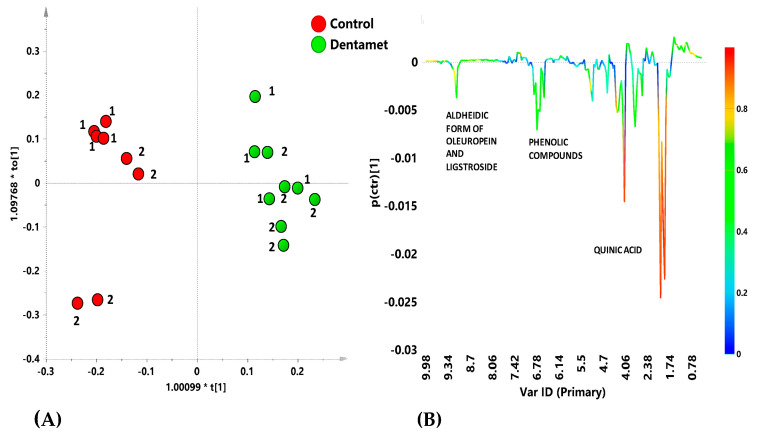
(**A**) Orthogonal partial least squares-discriminant analysis (OPLS-DA) score plot for Cellina di Nardò treated with Dentamet^®^ (green circles) and untreated (control) (red circles) leaf extracts samples (1 + 1 + 0; R2X = 0.567; R2Y = 0.957; Q2 = 0.897); (**B**) S-lineplot for the model, indicating molecular components responsible for the class separation. The corresponding predictive loadings are colored according to the correlation scaled loading [p(corr)]. Reproduced from [40].

The metabolic response of both infected and treated adult olive cultivars Ogliarola salentina and Cellina di Nardò was then investigated in two sampling periods performed during the first year of the treatments with Dentamet^®^ to the olive crown [40]. The ^1^H-NMR metabolomic approach, in conjunction with a multivariate statistical analysis, showed that for both these *X. f*. subsp. *pauca*-susceptible cultivars, some metabolites such as quinic acid, the aldehydic form of oleuropein, ligstroside and phenolic compounds, were consistently found as discriminative for the untreated trees (Figure 9 and Figure 10). Quinic acid, a precursor of lignin, was confirmed as a disease biomarker for the olive trees infected by *X. f.* subsp. *pauca*, as previously observed for the same cultivars [41,42] and for *X. f.* subsp. *fastidiosa*-infected grapevines [43]. The two cultivars showed a distinct response to the biocomplex treatment. A consistent increase in malic acid was observed for the Ogliarola salentina trees, whereas in the Cellina di Nardò trees, the treatments seemed to induce the accumulation of γ–aminobutyrate (GABA), as already described as a response to environmental strain and considered as an adaptive stress-mitigating factor [44,45]. 

Recently, a ^1^H-NMR metabolomic approach was used to analyze the xylematic extracts of the *X. f.* subsp. *pauca*-tolerant cultivar Leccino in comparison with the susceptible cultivars Ogliarola salentina and Cellina di Nardò following a mid-term period of Dentamet^®^ spray treatment (i.e., three years of spray treatments during spring and early autumn). The metabolic profiles evaluation focused in a specific time span (six months treatment April to September after six months suspension), with the aim to evaluate any possible memory effects due to previous treatments persisting after suspension periods. Therefore, metabolomic analyses were performed on leaf sample extracts from infected and untreated trees in two specific samplings (March, before the start and October after the end of treatments) over the last treatment year [46].

Statistical models, obtained for untreated and treated samples, respectively, demonstrated a different degree of separation, more pronounced in the second sampling, essentially related to the higher mannitol content in the treated samples. The intracellular accumulation of mannitol can be considered as a strategy to improve tolerance against water deficit thanks to its osmoprotectant and antioxidant ability to protect chloroplast [47,48]. Moreover, as already reported [49], the accumulation of mannitol was observed in olive leaves in response to foliar fertilization, suggesting an improvement of physiological performance and the photosynthetic capability of olive trees. For the untreated samples, a higher relative content of phenolic compounds such as tyrosol and hydroxytyrosol moieties of oleuropein and its aldehydic forms and quinic acid were observed in the second sampling for all the analyzed cultivars, consistent with previous findings [39,50]. The analyses of the specific influence of the sampling period on metabolic profiles for infected cultivars indicated a specific rank (i.e., Leccino > Cellina di Nardò > Ogliarola salentina) for the observed discrimination between the two sampling periods [46].

Moreover, an increasing intraclass discrimination between treated and untreated sample groups could be reported for all the cultivars in the second sampling, suggesting the occurrence of different levels of disease, season and treatment effects over the considered time span. A significant differentiation among treated and untreated samples also in the first sampling was observed only for Leccino, suggesting a possible memory effect of the previous year treatment. The comparison of tolerant Leccino with respect to the susceptible cultivars Ogliarola salentina and Cellina di Nardò, grouped as a single class, demonstrated a general smoothing of the observed differences in the second with respect to the first sampling for both treated and untreated samples.

**Figure 11:**
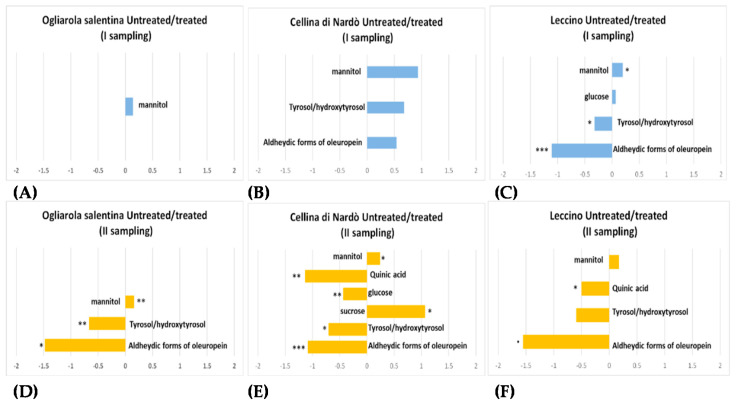
Magnitude scale of the levels of discriminating metabolites comparison between untreated and treated olive trees in two sampling periods (March and October). Ogliarola salentina (**A**,**D**), Cellina di Nardò (**B**,**E**) and, Leccino ((**C**,**F**)) cultivars provided as values of −Log2 (FC). Metabolites with −Log2 (FC) negative values have higher concentration in untreated samples, while −Log2 (FC) positive values indicated metabolites with higher concentration treated samples. Statistical significance (one-way analysis of variance (ANOVA)) was set at least at adjusted *p*-values < 0.05 and indicated with 0 ‘***’ 0.001 ‘**’0.01 ‘*’ ′0.05 ′. Reproduced from [46].

Higher amounts of flavonoids were observed both in infected untreated and treated Leccino cultivar. On the contrary, in the susceptible cultivars, variables ascribable to mannitol signals and indicating a higher relative sugar content with respect to Leccino were observed (Figure 11). The observed differences in the infected Leccino with respect to susceptible cultivars showed interesting reversed behavior for mannitol levels with respect to the analogous comparison for healthy trees (Table 1). Indeed, Leccino healthy leaf samples showed a higher content of mannitol and sucrose with respect to the healthy susceptible cultivars—Ogliarola salentina and Cellina di Nardò. The high constitutive sucrose content, observed for Leccino cultivar, could also be involved in the *X. f.* subsp. *pauca* resistance due to sucrose’s role as a signaling molecule in response to cavitation [42].

The overall results highlighted how the infection caused by *X. f.* subsp. *pauca* strongly modifies the metabolism of olive trees, and how a zinc-copper-citric acid biocomplex can induce an early reprogramming of the metabolic pathways in the infected trees. The different responses to Dentamet^®^ treatment would seem to be correlated to olive cultivars physiology and/or the pathogen attack levels. 

## 8. The Control Strategy at Work

In addition to the spraying of Dentamet^®^ onto the olive tree crown once per month from spring to autumn, the *X. f.* subsp. *pauca* control strategy employs agronomical measures to reduce the population of the insect vector (*Philaenus spumarius*) in surrounding vegetation and to maintain a regular shape of the olive tree crown by regular pruning. Maintaining or restoring good soil fertility is also important for the efficacy of the strategy. The vector density can reach very high levels in the spring, when 10 to 40 individual nymphs of *P. spumarius* can be found on each m^2^ of soil hosting wild plant species [51,52]. From the end of spring to early autumn, *P. spumarius* adults can reach the young olive leaves and release *X. f.* subsp. *pauca* directly into the leaf xylem. Mechanical removal of weeds by harrowing during this period reduces the risk of pathogen spread within and between the olive groves. This is enforced by the Apulia region officers, who impose a fine for failure to remove weeds at the end of winter into spring.

Regular pruning of the olive tree every one to two years maintains an equilibrium between vegetative growth and fruit production, and facilitates pest control and harvest [53,54]. The distribution of a foliar spray such as of Dentamet^®^ is facilitated by a well-aerated canopy, and thorough coverage of the leaves augments the efficacy of the treatment. Hard pruning, performed on a four- to five-year basis, was the norm during the last decades in most of the Salento olive groves, but this resulted in shading, a haphazard spatial distribution of the canopy, shorter and more compact vegetative growth, and deadwood in the inner part of the tree, ultimately leading to decreased tree longevity and lower productivity [55,56]. Contrary to an effective reduction of *X. f*. subsp. *pauca* density within citrus trees obtained in Brazil with pruning [57], the attempts to reduce the bacterium inoculum within olive trees of Salento through severe pruning caused the death of the tree during the following months [33,56]. Thus, light pruning aiming at maintaining just a physiological equilibrium of the tree between the vegetative and productive phases should be now performed in Salento.

Maintenance of good fertility and beneficial microbial communities in the soil is important in pathogen control [58]. Depletion of zinc, copper, and manganese content has been observed in the soil of Salento, where the OQDS symptoms are very severe. Deficiency in these ions was also observed in the leaves of diseased olive trees that grow in this area [59,60]. In areas north of the infected zone (i.e., north of the Bari areas), a higher zinc and copper content both in soil and olive leaves is found in spite of the lithological matrix (calcareous soils) being similar in the two areas [59]. Depletion of zinc and copper in the Salento soil may explain the effectiveness of Dentamet^®^ in restoring good vegetative growth, since zinc and copper are involved in many plant metabolic pathways other than plant defense mechanisms [61,62]. Apart from micronutrients formulations, beneficial microorganisms are also important for augmenting resistance to *X. f.* subsp. *pauca* [33].

## 9. A Novel Endotherapy to Fine-Tune the Tree Recovery

Bacterial pathogens on the leaf surface of plants can be effectively controlled by direct contact with copper applied by foliar sprays [63,64]. With xylem-invading pathogens such as *Xylella fastidiosa*, the antimicrobial compounds must contact the pathogen within the xylem vessels. This is more likely to occur through injection than through foliar application or soil drenching [65]. Delivery of antimicrobials by injection into the plant’s vascular system (endotherapy) offers multiple advantages compared to foliar applications, especially in diseases such as olive quick decline syndrome that are caused by xylem-restricted pathogens. Endotherapy enhances the effectiveness of the treatment by delivering the compound directly to the pathogen, and accelerates translocation of the injected antimicrobial compounds, even in heavily infected plants with limited remaining foliage. Endotherapy avoids the unintended dissipation of the antimicrobial compound into the air and the broader ecosystem (closed application system) and therefore significantly improves worker and bystander safety by minimizing dermal and inhalatory exposure (https://www.epa.gov/pesticide-science-and-assessing-pesticide-risks/occupational-pesticide-handler-exposure-data#phed). Finally, endotherapy reduces the amount of active ingredients that are applied and reduces the overall environmental load when compared to foliar application methods.

The use of traditional endotherapy methods has been of limited use due to several important constraints. The primary constraint is that many systems are based on drilling sizable holes in the trunk (4 to 12 mm, 5 to 120 mm depth) [66,67], leading to significant plant damage and potential entry points for secondary infections with other pathogens [68]. Notably, repeated treatments of infected plants can lead to a negative impact on tree health due to less effective wound closure and compartmentalization [69,70]. A second important constraint is that the formulated active ingredient is delivered to the heartwood of the trunk, which presumably leads to a slower distribution of the active ingredient(s) throughout the plant. The heartwood has no or at least a limited physiological role, in contrast to the active xylem, which transports water and nutrients throughout the plant [71]. Additionally, it was observed that the sap flux decreases significantly towards the heartwood boundary [72]. In addition, the traditional systems have operational limitations limiting the number of trees that can be treated by one person to fewer than 100 per day. 

**Figure 12:**
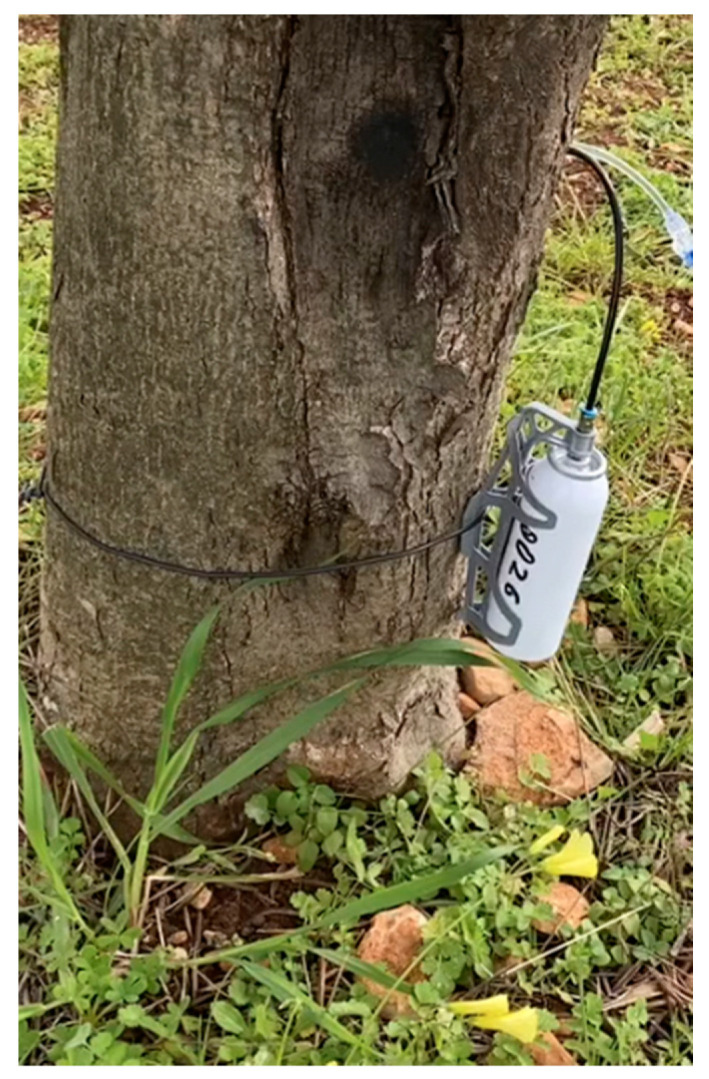
Prototype of Invaio Sciences TIPS^TM^ injection system applied to an olive tree. The system allows a low-pressure release of compounds.

**Figure 13:**
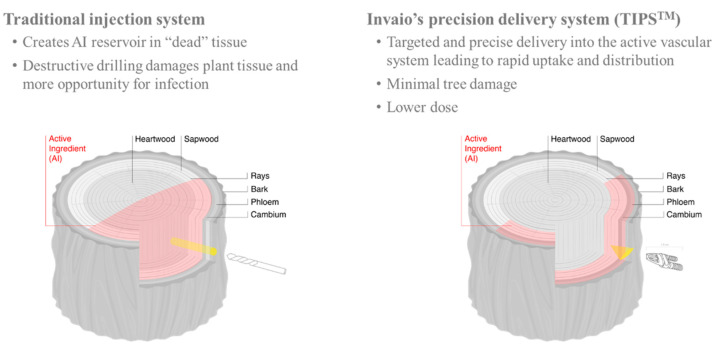
Comparison traditional injection system and Invaio’s injection system (based on internal Invaio Sciences data and insights).

In July 2020, a proprietary injection system produced by Invaio Sciences was used to inject 30 trees in the Salento area with a micronutrient containing zinc and copper ions. The Invaio system includes an extremely thin, arrowhead-shaped injection tip, resulting in minimal invasiveness and plant damage (Figure 12 and Figure 13). The tip is only 12 mm long and 1.5 mm thick, with a size and shape designed for the precise and effective delivery of treatment formulations to the tree’s active vasculature. The formulations are injected at low pressure (between 1 and 3 bars) directly into the vascular system, thus enhancing uptake and distribution dynamics of the injected compound. The injection system and its operating parameters enable a higher throughput of injections compared to traditional systems. Furthermore, the tip is designed to avoid clogging, allowing efficient delivery. We are also investigating whether multiple injections with the same tip are possible.

## 10. Considerations and Perspectives

A great part of the Salento territory has been facing a dramatic decline of olive health during this last decade [25]. Records of OQDS symptoms document the spread of *X. f.* subsp. *pauca* from its initial invasion in the Gallipoli area about from 2008 [22], so that within barely 15 years, a multi-millennial agro-ecosystem is at risk of extinction, with a subsequent impoverishment of a historical, social, economic, and landscape heritage [26].

In recent years, we have shown that a coexistence with *X. f.* subsp. *pauca* is possible in the so-called “infected area” of Salento (i.e., the province of Lecce, and part of the provinces of Brindisi and Taranto). The tree crown nebulization of Dentamet^®^ (a biocomplex containing zinc, copper, and citric acid) during spring, summer, and early autumn significantly reduced both the symptoms (i.e., twig and branch wilting) and the bacterial cell density within the leaves of the sensitive olive cultivars Ogliarola salentina and Cellina di Nardò as measured by quantitative real-time PCR [33,34]. Dentamet^®^ treatments resulted in a rapid reprogramming of some indicative metabolic pathways, as observed by metabolomics, which re-established a more normal tree physiology [39,40,46]. The yearly amount of copper released in the environment by means of this control strategy satisfies the current requirements aimed at avoiding the accumulation of copper in the soil [73]. It has been calculated that the amount of copper and zinc released in the soil in one year by the six treatments is 0.06 ppm and 0.12 ppm, respectively. Finally, an innovative endotherapy approach appears to provide a more efficient technique for promoting regrowth in severely damaged trees and reducing the amount of product required to treat a tree and thus reducing the cost of the control strategy as well.

**Figure 14:**
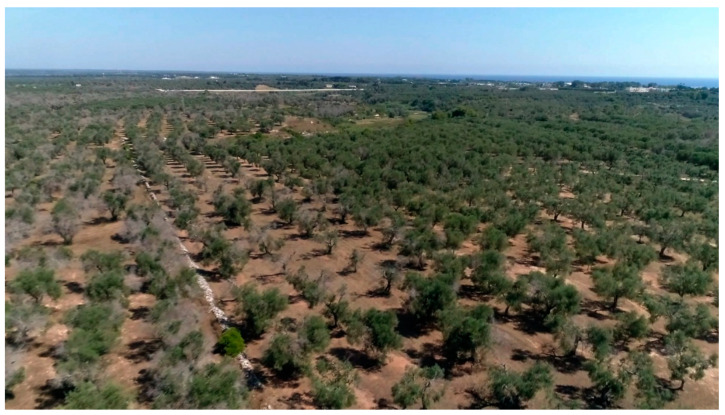
A comparison between a non-treated (on the left) and treated olive grove (on the right) located in the Otranto area (Lecce province; coordinates: Lat. 40.138095; Lon. 18.467825) within the “infected” area. It is evident to see the capability of tree restoration provided by the application of the control strategy.

**Figure 15:**
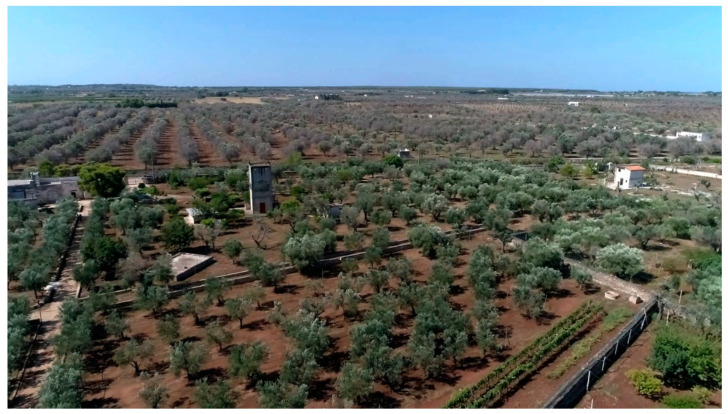
An olive grove located in Nardò (Lecce province; coordinates: Lat. 40.187637; Lon. 17.987413) that, since 2016, has been following the control strategy described in the text. It is located in the “infected” area and borders a territory with dead or very damaged olive trees.

Collectively, these results demonstrate an efficient, simple, low-cost, and environmentally sustainable strategy to control *X. f.* subsp. *pauca* in Salento, at least in the areas not already completely destroyed by the bacterium. The complete restoration of the olive agroecosystem is no longer possible, but the application of the measures herein described on a large scale could allow some preservation of it, without sacrificing the typical Apulian olive growing cultivars, such as Cellina di Nardò and Ogliarola salentina. Despite the general opinion that it was not possible to control the bacterium, a number of farmers of the provinces of Lecce, Brindisi, and Taranto are applying the control strategy herein described, this representing valid examples of resilience for the territory. Within this concept, in Salento, Slow Food has promoted two clusters of resilient farmers to enhance their work and attest to their perseverance in working to preserve the local heritage. Apart from this initiative, currently, there are more than 700 ha of olive groves in the provinces of Lecce, Brindisi, and Taranto where this strategy is being applied to control *X. f*. subsp. *pauca* (Figure 14 and Figure 15).

## Figures and Tables

**Table 1:** Main metabolites identified through ^1^H-NMR metabolomic analyses as discriminating in leaf extracts samples of olive cultivars Ogliarola salentina, Cellina di Nardò and Leccino naturally infected by *X. f.* subsp. *pauca*, treated with Dentamet^®^ or healthy. Based on [39,40,46]. Note quinic acid as a biomarker for naturally infected trees, and mannitol for treated and healthy Leccino trees.

	Infected						Healthy		
	*Ogliarola*		*Cellina*		*Leccino*		*Ogliarola*	*Cellina*	*Leccino*
	Untreated	Treated	Untreated	Treated	Untreated	Treated	Untreated	Untreated	Untreated
**Oleuropein** **derivatives**	✓		✓	✓	✓		✓	✓	
**Glucose**	✓		✓						
**Sucrose**									✓
**Flavonoids**						✓			
**Mannitol**		✓		✓		✓			✓
**Malate**		✓							
**Quinic acid**	✓		✓		✓				

## Data Availability

No new data were created or analyzed in this study. Data sharing is not applicable to this article.
